# The Nursing Club in Dublin

**Published:** 1919-10-25

**Authors:** 


### IRISH COLLEGE FORGING AHEAD.

### The Nursing Club in Dublin.

The College of Nursing Club in Dublin is a *fait accompli.* The idea was conceived by Miss Matheson, and in the face of very substantial difficulties it has been established. The premises are situate in the best residential quarter of the city. A few years ago Fitzwilliam Square was a centre of fashion, but owing to the motor-car it is largely bereft of its former splendour, and is now inhabited chiefly by medical men and other professional people who find it necessary to live in the midst of their work. No. 5a is an attractive, airy building with lofty apartments and a particularly bright outlook. Within easy walking distance of the principal hospitals, the Club bids fair to be a most successful enterprise.

The scheme of membership is well designed. All trained nurses and midwives are eligible, whether they belong to the College or not, and probationers will be especially welcome, although they will not be full members until their training is complete. Members of the College of Nursing will pay an annual subscription of five shillings, and non-members half a guinea. Nurses passing through Dublin, or visiting the city for a short stay, will here find comfortable sleeping accommodation and board for a modest charge. When the house is completely furnished the Club will be an invaluable institution. It will provide what working nurses want above all other things—a home.

### A Difficult Problem.

The difficult problem of providing funds now remains to be solved. The Club will never be self-supporting in the best of circumstances, and for the obvious reason that the profession in Dublin is too wretchedly paid. The public owe a tremendous debt to the nurse, and are now going to be asked to pay an instalment. From £1,200 to £1,500 is necessary to give it a fair start, and the first step towards finding this sum was taken on Friday of last week, when a small but very influentially attended meeting was held in the Club. The Lord Chief Justice was present, and showed a keen interest in the proceedings. Sir John Lynch, a prominent citizen, and a Governor of many Hospitals, was equally enthusiastic, and offered much helpful advice. Sir John Lumsden, K.B.E., presided, and with the co-operation of Dr. George Peacocke, Chairman of the Irish branch of the College, set forth the aims and ambitions of the new Club. The chief point, strongly accentuated, was that the Club members, whether they belong to the College or not, will have equal rights, and that in its management the democratic note will prevail. The Lord Chief Justice proposed that before appealing to the Irish public for funds a constitution should be drawn up showing that all nurses are welcome, and that there will be a complete absence of cliques and factions. With this suggestion the meeting was in unanimous accord. The result was the formation of a strong Committee, whose functions will consist of raising the necessary funds, drawing up the suggested constitution, and of propaganda. Miss Hignett, O.B.E., a member of this Committee, proposed the holding of a money-producing function in the Club premises soon after Christmas. This was warmly approved, and the services of many helpers were enlisted. Miss Matheson and Miss Harvest, Secretary of the Club, will, of course, be members of this Executive Committee.

### What the Club Stands for.

The Club stands for nurse comradeship, but the difficulties in the way of producing this desirable quality are as great as they are hard to understand. The chief obstacle in the way is the active band of Pussyfoot campaigners who have always allied themselves with retrogression. Their opposition may be counted upon, for obstruction is the breath of their nostrils. When the English Midwives Bill was passing through Parliament they worked with frenzied zeal to prevent its application to Ireland, and it was only when Ireland became the dumping ground for English and Scottish Sairey Gamps, driven out of office in their own countries, that they realised their stupidity. When Sir Arthur Stanley initiated the Nation's Tribute to Nurses they derided it as an appeal for charity. Sir Arthur came to Ireland, and by his personal magnetism and influence gathered together a band of workers who collected £10,000 for the benefit of nurses unemployed, aged, and infirm. He addressed and inspired large meetings in Dublin and Belfast. But all the time Pussyfoot was poisoning the springs, and when success was in sight the instigator of the movement was politely informed that neither he nor the College were to be permitted to have anything further to say to it.

I quote these things because so far as the enemies of the nurse referred to are concerned the situation is unchanged. They have nothing to their credit by way of achievement, and, so far as I have been able to observe, the policy of the College has been to placate them and make them reasonable. It is a mistake. There is one only way, and that is to fight them and to smite them. They have already robbed the College of the fruit of its labour in the matter of the Tribute. They will try, if possible, to wreck the Club. I know them, and if the College intends to win it will have to become the College militant. Vigorous propaganda in the Press, by leaflet, and by personal contact must be undertaken. Otherwise, with means of hymns of hate, winks, nods, and innuendos, Pussyfoot will spoil the fabric before its value and utilities become known.

